# Probiotics and Prebiotics in Subclinical Hypothyroidism of Pregnancy with Small Intestinal Bacterial Overgrowth

**DOI:** 10.1007/s12602-023-10068-4

**Published:** 2023-04-10

**Authors:** Qian Ouyang, Yajuan Xu, Yanjie Ban, Jingjing Li, Yanjun Cai, Bo Wu, Yingqi Hao, Zongzong Sun, Miao Zhang, Mengqi Wang, Wentao Wang, Yinkai Zhao

**Affiliations:** Department of Obstetrics and Gynecology, The Third Affiliated Hospital of Zhengzhou University, Zhengzhou, China

**Keywords:** Subclinical hypothyroidism during pregnancy, Small intestinal bacterial overgrowth, Probiotics, Prebiotics, Treatment

## Abstract

Evaluating efficacy of probiotics combined with prebiotics in small intestinal bacterial overgrowth (SIBO) in subclinical hypothyroidism (SCH) in the second trimester. We collected data from 78 pregnant women with SCH (SCH group) and 74 normal pregnant women (control group) in second trimester, compare the differences in high sensitivity C-reactive protein (hsCRP), result of lactulose methane-hydrogen breath test and gastrointestinal symptoms assessed by GSRS scale between two groups. In SCH group, 32 patients with SIBO were selected as intervention group. Treatment with probiotics + prebiotics for 21 days; The differences of lipid metabolism, hsCRP, thyroid function level, methane-hydrogen breath test results and GSRS scores before and after treatment were compared to evaluate the therapeutic effect. (1) The positive rate of SIBO and methane, hsCRP levels in SCH group were higher than those in control group (*P* < 0.05), the total score of GSRS scale, mean score of indigestion syndrome, and constipation syndrome in SCH group were higher (*P* < 0.05). (2) The mean abundance of hydrogen and methane were higher in SCH group. (3) After treatment, serum levels of thyrotropin(TSH), total cholesterol(TC), triglyceride(TG), low-density lipoprotein (LDL), and hsCRP in intervention group were decreased, and high-density lipoprotein (HDL) was increased compared with before treatment (*P* < 0.05). (4) After treatment, methane positive rate, total score of GSRS scale, mean score of diarrhea syndrome, dyspepsia syndrome, and constipation syndrome were decreased (*P* < 0.05). (5) The average abundance of methane and hydrogen were lower. Probiotics combined with prebiotics are effective in the treatment of SIBO in pregnant SCH patients.

Clinical Trial Registration Number: ChiCTR1900026326.

## Introduction

Subclinical hypothyroidism during pregnancy(SCH) refers to thyroid-stimulating hormone (TSH) in pregnant women that is higher than the upper limit of the reference value, and free tetraiodothyronine (FT_4_) is in the reference value; the prevalence ranges from 2.0 to 2.5% [1, 2]. Studies have shown that the incidence of spontaneous abortion, placental abruption, and premature rupture of membranes in pregnant women with SCH is higher than that in normal pregnant women [3, 4]. In addition, SCH during pregnancy is associated with low birth weight infants and small for gestational age infants, which can lead to lower IQ and psychomotor ability of offspring, and increase the risk of cardiovascular defects [5], seriously affecting the health of mother and child.

In recent years, the intestinal flora has become a current research hotspot. Teng et al. [6] indicated that changes in thyroid ho meostasis will affect the homeostasis of gut microbiota, disturbances of gut microbiota will further lead to an imbalance of thyroid homeostasis, and the two influence each other. Knezevic et al. [7] believe that the bacterial overgrowth associated with hypothyroidism is mainly related to the small intestine, decreased intestinal nerve motility in patients with hypothyroidism, and edema of the intestinal muscularis, which can lead to bacterial overgrowth in the small intestine. The methane-hydrogen breath test is a noninvasive test that has emerged in recent years to determine bacterial overgrowth in the small intestine. At present, there are few studies on probiotics and prebiotics in the treatment of intestinal bacterial overgrowth, and their specific efficacy is not supported by evidence [8]. A study has shown that the combination of probiotics and prebiotics has a stabilizing effect on hypothyroidism [9].Chen et al. in 2020 [10] suggested that the quadruple probiotics combination consisting of *Bifidobacterium infantis*, *Lactobacillus acidophilus*, *Enterococcus faecalis*, and *Bacillus cereus* has anti-inflammatory effects and can help repair the damaged intestinal barrier. Recent studies have shown that multi-strain probiotics may be more effective than single strain probiotics, and dietary fiber, which belongs to prebiotics, can be beneficial to health by changing the gastrointestinal microflora [11, 12]. This study explored the positive effects of probiotics combined with prebiotics on thyroid function control and small intestinal bacterial overgrowth in patients with SCH complicated with SIBO in pregnancy based on the treatment of levothyroxine sodium (LT4) to provide new ideas for the treatment of subclinical hypothyroidism complicated with SIBO in pregnancy.

## Experimental Method

### Study Subjects

Clinical Trial Registration Number: ChiCTR1900026326. Name: Study of intestinal bacterial overgrowth and complications during pregnancy. Participants: Seventy-eight pregnant women with subclinical hypothyroidism were selected as SCH group, 74 normal pregnant women were selected as Control group, and SCH group who combined with intestinal bacterial overgrowth were selected as the intervention group. All subjects signed informed consent.

### Inclusion Criteria

(1) Diagnosis of hypothyroidism during pregnancy: The regional reference value range of thyroid function recommended by our hospital in accordance with guidelines (commercial kit, Roche, Shanghai, China) [1, 13]: 11.5 < FT4 < 22.7 pmol/l,TSH ≥ 4.2mIU/L; control group: 11.5 < FT4 < 22.7 pmol/l, 0.4 < TSH < 4.2mIU/l. (2) No other pregnancy complications. (3) The pregnant women with SCH had good control of thyroid function, and the TSH level had been maintained below the treatment target of 2.5mIU/L; (4) All participants were Han Chinese who lived in Zhengzhou, Henan Province, China, and had a similar diet.

### Exclusion Criteria

(1) Those with endocrine-immune pregnancy complications, such as diabetes, hypertension, systemic lupus erythematosus, and abnormal thyroid function found before pregnancy. (2) Women with multiple pregnancies. (3) Artificial insemination or assisted reproductive technology to assist pregnancy. (4) Pregnant women with a history of adverse pregnancy and childbirth. (5) Pregnant women with positive thyroid peroxidase antibody. (6) Use of probiotics, prebiotics, antibiotics, or other drugs that affect the intestinal flora in the past 3 months.

## Research Methods and Observation Indicators

### Lactulose Methane-Hydrogen Breath Test

The specificity of the lactulose methane hydrogen breath test is higher than that of the glucose methane hydrogen breath test [14]. All included subjects were required to abstain from taking any antibiotics for the first 4 weeks prior to the methane-hydrogen breath test and followed these conditions the day before:roasted or broiled chicken, fish, turkey (salt and pepper only), white bread (white bread only), steamed white rice, eggs, white water chicken, or beef broth, drink only unsweetened water (for 1 full day), continue taking levothyroxine sodium tablets (according to physician instructions), and wash one’s mouth after getting up on the same day.

Breath samples were collected using a mouthpiece device BST 20 mm, 25 mm, and 30 mm (Aibos Medical Technology, Beijing, China). After fasting, the frist sample was blown on an empty stomach to set for 0 h, and 15 ml of lactulose (Dumic, China) was then orally added as a 10% aqueous solution (lactulose 15 g + warm water 50 ml) within 10 s. New breath samples were collected at 20, 40, 60, 80, and 100 min after ingestion of lactulose.

Hydrogen (H_2_) and methane (CH_4_) levels in the samples were simultaneously measured by gas chromatography using a QuinTron MicroLyzer Model 12i (QuinTron Instruments, Milwaukee, WI, USA). The principle of the test is that the normal intestine contains four kinds of gases: (1) swallowed air; (2) gas mixed with food; (3) combination reaction in the intestine and gas diffusion in the blood; (4) gas production by intestinal microorganisms. The average intestinal gas of healthy subjects is 100 mL,including hydrogen (H_2_), methane (CH_4_), ammonia (NH_3_), carbon dioxide (CO_2_), nitrogen (N_2_), hydrogen sulfide (H_2_S), and indol, while microorganisms produce H_2_ and CH_4_ [15].

Small intestine bacterial overgrowth (SIBO) commonly defined as 10^5^ or more colony-forming unitsper milliliter (CFU/mL) of bacteria grown from the intestinal suction [15]. Based on hydrogen and methane breath tests in gastrointestinal diseases [9], the results of breath tests based on the North American Consensus are analyzed below [15]: (1) Hydrogen was positive when the hydrogen abundance within 90 min rises ≥ 20 ppm from the baseline value; (2) Methane was positive when within 90 min, the methane abundance rises by ≥ 10 ppm from the baseline value; (3) Because methanogens consume hydrogen to produce methane, in order to improve the accuracy of breath test, the standard of the manufacturer of methane and hydrogen breath test equipment is set (hydrogen + methane) was positive when if the hydrogen and methane abundances do not reach the above values, the sum of the two is higher than the sum of the hydrogen + methane baseline values, and the methane abundance is greater than 15 ppm within 90 min. One of the three must meet the requirement that SIBO be positive. (Since the duration of blindness in Asian population is shorter than that in Westerners and lactulose will shorten the duration of oral blindness, this study adopted 20-min intervals of air blowing to reduce the false positive rate).

### Clinical Symptoms

The Gastrointestinal Symptom Rating Scale (GSRS) is a disease-specific tool consisting of 15 items, each of which is rated on a seven-point Likert scale, with one point indicating no discomfort and seven point indicating very severe discomfort [16]. The 15 items of the GSRS scale were divided into five symptom clusters: abdominal pain (epigastric pain, hunger pain, and nausea); reflux syndrome (heartburn and acid reflux), diarrhea syndrome (diarrhea, loose stools, and urgent defecation), indigestion syndrome (abdominal noise, abdominal distention, burping, and increased exhaust), and constipation syndrome (constipation, hard stools and straining and feeling of inactivity). The average score of each symptom cluster was obtained by averaging the individual scores of all items in the symptom cluster [17].

## Intervention Methods

The SCH group and control group underwent lactulose methane-hydrogen breath tests, respectively, and gestational serum was collected on an empty stomach in the morning of the same day to measure hsCRP levels. The intervention group was given probiotics and prebiotics; 21 days as a course of treatment, the intervention group received lactulose methane-hydrogen breath test again after one course of treatment. And gestational serum was collected on an empty stomach in the morning of the same day to measure thyroid function, blood lipid level, and hsCRP level.

### Probiotics

Quadruple probiotics (Siliankang, national drug approval number: S20060010),which is composed of intestinal probiotics, is a compound preparation, the main ingredients include: *Bifidobacterium infantis* 2.7 × 10^8^ CFU/g, *Lactobacillus acidophilus* 4.7 × 10^8^ CFU/g, *Enterococcus faecalis* 6.1 × 10^7^ CFU/g, *Bacillus cereus* 1.5 × 10^6^ CFU/g. Usage:1.5 g, 3 times a day.

### Prebiotics

Polysaccharide fiber powder (Risicom ^®^, production license No: SC13061011200721) is a dietary supplement. This product is composed of a variety of dietary fiber, the main components include inulin, ice sugar, microcrystalline cellulose, and oat fiber. Usage:5 g, 3 times a day.

## Statistical Analysis

Statistical analysis was performed using SPSS 26.0 Software, and the data were imaged using GraphPad Prism 8 (GraphPad Software, San Diego, USA). Continuous data are described as the means ± standard deviation, and count data are expressed as the frequency and rate. Two independent samples that conformed to a normal distribution and homogeneity of variance were analyzed by *t* test, corrected *t* test, and paired-sample *t* test. Those that do not conform to normal distribution are tested by rank sum test, paired sample rank sum test. The comparison of count data between two independent samples and binary data was analyzed by the *X*^2^ test and corrected *X*^2^ test.

## Result

**Fig. 1:**
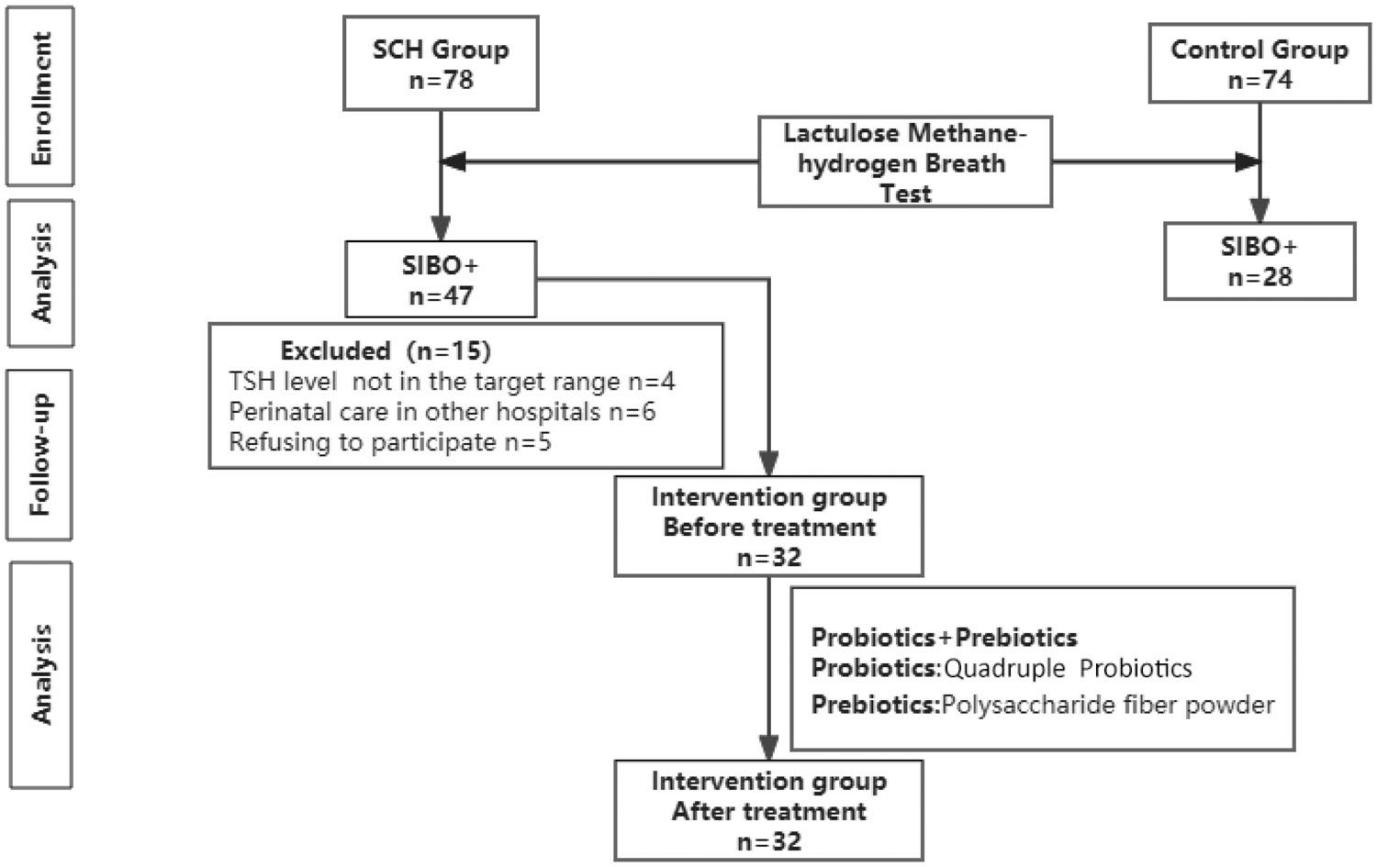
Data on pregnant women with subclinical hypothyroidism and with normal condition

A total of 78 pregnant women with subclinical hypothyroidism in the second trimester and 74 pregnant women with normal conditions were enrolled at the start of the study, and 32 of 47 pregnant women with subclinical hypothyroidism with SIBO completed the trial. Six subjects received perinatal care in other hospitals, and 5 subjects refused to participate in the follow-up trial were excluded. In addition, 4 subjects whose TSH levels were not maintained within the target range were excluded (Fig. 1).

**Fig. 2:**
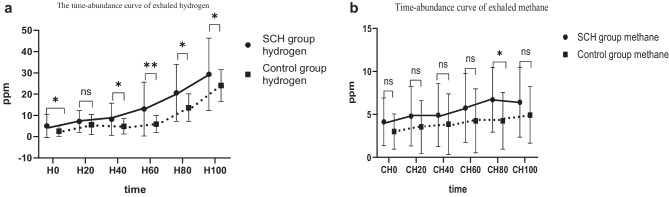
**a** Mean abundances of hydrogen (ppm) in breath samples collected at 0, 20, 40, 60, 80, and 100 min after fasting and after lactulose ingestion in the SIBO of SCH group (*n* = 47) and SIBO of the control group (*n* = 28). **b** Mean abundances of methane (ppm) in breath samples collected at 0, 20, 40, 60, 80, and 100 min after fasting and after lactulose ingestion in the SIBO of SCH group (*n* = 47)and SIBO of the control group (*n* = 28).*p < 0.05 was considered statistically significance,**p < 0.001 was considered statistically significance, ^ns^ *p* > 0.05 was no statistical significance

**Fig. 3:**
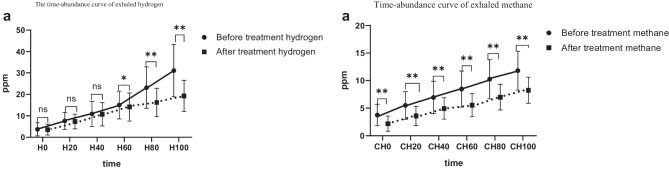
**a** Mean hydrogen abundance (ppm) in respiratory samples collected at 0, 20, 40, 60, 80, and 100 min after fasting and after lactulose ingestion in the intervention group after treatment (*n* = 32) and before treatment (*n* = 32). **b** Mean abundances of methane (ppm)in breath samples collected at 0, 20, 40, 60, 80 and 100 min after fasting and after lactulose ingestion from the after treatment group (*n* = 32) and before treatment group (*n* = 32).*p < 0.05 was considered statistically significance,**p < 0.001 was considered statistically significance, ^ns^ *p* > 0.05 was no statistical significance

1. This study included 78 patients in the SCH group and 74 patients in the control group during the same period. The general data of the two groups of subjects (Table 1) were as follows: the SCH group and the control group had no significant difference in age, gestational age, BMI, or gravidity (*P* > 0.05). The positive rate of lactulose methane-hydrogen, GSRS scale score and hsCRP in both groups (Table 2) were as follows: The positive rate of lactulose methane-hydrogen (60.3% vs. 37.8%) and positive rate of methane (17.9% vs. 6.8%) in the SCH group was higher than that in the control group, the differences were statistically significant (*P* < 0.05), there was no statistical significance in the positive rate of hydrogen and (hydrogen + methane) (*P* > 0.05). The level of hsCRP in SCH group (7.47 ± 2.57 vs. 3.84 ± 1.81) was higher than that in control group, the differences was statistically significant (*P* < 0.001). Total GSRS scale score of the SCH group (18.17 ± 2.90 vs.16.81 ± 2.03), mean score for indigestion syndrome (1.28 ± 0.51 vs. 1.09 ± 0.31) and mean score for constipation syndrome (1.38 ± 0.47 vs. 1.20 ± 0.46) was higher than that in the control group, the differences were statistically significant (*P* < 0.05); there was no significant difference in the average scores of abdominal pain, reflux syndrome, and diarrhea syndrome (*P* > 0.05).
2. Through lactulose methane-hydrogen breath test, the hydrogen abundance and methane abundance in the SIBO of SCH group were higher than those in the SIBO of control group at the same time, and the differences were statistically significant at 0, 40, 60, 80, and 100 for hydrogen, and at 80 for methane (*p* < 0.05) (Fig. 2).
3. The blood value of the intervention group was tested before and after probiotics and prebiotics treatment (Table 3): thyroid function level: TSH level (2.46 ± 0.57 vs. 1.63 ± 0.38) was lower after treatment than before treatment, and the difference was statistically significant (*P* < 0.001), there was no significant difference in FT4 level before and after treatment (*P* > 0.05). Blood lipid level: after treatment, TC (5.45 ± 1.24 vs. 3.35 ± 0.74), TG (2.25 ± 1.48 vs. 1.26 ± 0.84), and LDL (2.96 ± 0.87 vs. 1.81 ± 0.48) decreased compared with before treatment (*P* < 0.001), and the level of HDL (1.87 ± 0.27 vs. 2.83 ± 0.37) increased after treatment compared with before treatment, and the difference was statistically significant (*P* < 0.001). hsCRP level: The hsCRP level after treatment was lower than that before treatment (7.60 ± 1.28 vs. 4.25 ± 1.08), the difference was statistically significant (*P* < 0.001). There was no significant difference in free fatty acid (NEFA), apolipoprotein A (ApoA), and apolipoprotein B (ApoB) before and after treatment (*P* > 0.05).
4. For the intervention group, the GSRS scale scores and lactulose methane-hydrogen breath test result before and after treatment were as follows (Table 4): Total GSRS scale score after treatment (20.41 ± 3.20 vs. 17.19 ± 1.65), diarrhea syndrome average score (1.26 ± 0.58 vs. 1.13 ± 0.33), indigestion syndrome average score (1.59 ± 0.67 vs. 1.17 ± 0.29) and constipation syndrome average score (1.46 ± 0.65 vs. 1.17 ± 0.28) lower than before treatment, the difference was statistically significant (*P* < 0.05). Lactulose methane-hydrogen breath test: The negative conversion rate was 28.1% after treatment, and the positive rate of methane was lower after treatment than before treatment (34.4% vs. 12.5%), the difference was statistically significant (*P* < 0.05), there were no significant differences in the positive rate of hydrogen and (hydrogen + methane) before and after treatment (*P* > 0.05).
5. After lactulose methane-hydrogen respiration test, the abundance of hydrogen and methane in the intervention group was lower than that before treatment. The difference of hydrogen at 60, 80, and 100 time points was statistically significant, and the difference of methane at 0, 20, 40, 60, 80, and 100 time points was statistically significance (*p* < 0.05) (Fig. 3).

**Table 1:** Comparison of general conditions between the SCH group and the control group

	SCH group (*n* = 78)	Control (*n* = 74)	*P* value
Maternal age, year*	30.46 ± 4.64	31.26 ± 4.55	0.288
BMI kg/m^2^*	22.75 ± 2.31	23.39 ± 3.19	0.156
Gravidity, n*	2.10 ± 0.93	2.20 ± 1.26	0.580
Parity, n*	0.55 ± 0.55	0.51 ± 0.63	0.693
Gestational age, week*	19.56 ± 3.39	19.19 ± 3.35	0.163

*Data are expressed as the means ± standard deviations using Student’s *t* test. *p* < 0.05 was considered statistically significance

*BMI* body mass index

**Table 2:** Comparison of the methane-hydrogen positive rate, GSRS scale score and hs-CRP between the SCH and control groups

	SCH group (*n* = 78)	Control (*n* = 74)	*P* value
**Lactulose methane-hydrogen breath test**			
Positive, *n* (%)	47 (60.3%)	28 (37.8%)	***0.006***^***a***^
Hydrogen +, *n* (%)	25 (32.1%)	19 (25.7%)	0.386^a^
Methane +, *n* (%)	14 (17.9%)	5 (6.8%)	***0.037***^***a***^
(Hydrogen + methane) +, *n* (%)	8 (10.3%)	4 (5.4%)	0.268^a^
**GSRS scale score**			
Total^***b***^	18.17 ± 2.90	16.81 ± 2.03	***0.001***
Abdominal pain^***b***^	1.13 ± 0.22	1.13 ± 0.28	0.864
Reflux syndrome^***b***^	1.04 ± 0.16	1.03 ± 0.11	0.441
Diarrhea syndrome^***b***^	1.15 ± 0.42	1.13 ± 0.40	0.774
Indigestion syndrome^***b***^	1.28 ± 0.51	1.09 ± 0.31	***0.007***
Constipation syndrome^***b***^	1.38 ± 0.47	1.20 ± 0.46	***0.02***
hsCRP*	7.47 ± 2.57	3.84 ± 1.81	**< *0.001***

^a^*X*^2^ test

^b^Data are expressed as the means ± standard deviations using sample rank sum test

*Data are expressed as the means ± standard deviations using Student’s *t* test, *p* < 0.05 was considered statistically significance

**Table 3:** Blood test level of the intervention group before and after treatment

Parameter	Before treatment (*n* = 32)	After treatment (*n* = 32)	*P* value
FT_4_ (pmol/l)*	13.03 ± 1.45	13.46 ± 1.49	0.176
TSH (μmol/l)*	2.46 ± 0.57	1.63 ± 0.38	**< *0.001***
TC (mmol/l)*	5.45 ± 1.24	3.35 ± 0.74	**< *0.001***
TG (mmol/l)*	2.25 ± 1.48	1.26 ± 0.84	**< *0.001***
HDL (mmol/l)*	1.87 ± 0.27	2.83 ± 0.37	**< *0.001***
LDL (mmol/l)*	2.96 ± 0.87	1.81 ± 0.48	**< *0.001***
FFA (mmol/l)*	0.37 ± 0.12	0.34 ± 0.12	0.062
ApoA (mmol/l)*	2.09 ± 0.56	1.93 ± 0.72	0.067
ApoB (mmol/l)*	0.99 ± 0.37	0.96 ± 0.35	0.057
hsCRP (mg/l)*	7.60 ± 1.28	4.25 ± 1.08	**< *0.001***

*Data are expressed as the means ± standard deviations using paired *t* test; *P* < 0.05 was considered statistically significance

**Table 4:** Comparison of GSRS score and lactulose methane-hydrogen breath test before and after treatment in intervention group

	Before treatment (*n* = 32)	After treatment (*n* = 32)	*P* value
**GSRS scale score**			
Total^a^	20.41 ± 3.20	17.19 ± 1.65	**< *0.001***
Abdominal pain^a^	1.23 ± 0.29	1.17 ± 0.25	0.083
Reflux syndrome^a^	1.11 ± 0.25	1.08 ± 0.18	0.157
Diarrhea syndrome^a^	1.26 ± 0.58	1.13 ± 0.33	***0.045***
Indigestion syndrome^a^	1.59 ± 0.67	1.17 ± 0.29	**< *0.001***
Constipation syndrome^a^	1.46 ± 0.65	1.17 ± 0.28	***0.001***
**Lactulose methane-hydrogen breath test**			
Positive, *n* (%)^2^	32	23 (71.9%)	***-***
Hydrogen +, *n* (%)^2^	16 (50.0%)	15 (46.8%)	0.802
Methane +, *n* (%)^2^	11 (34.4%)	4 (12.5%)	***0.039***^***b***^
(Hydrogen + methane) +, *n* (%)^2^	5 (15.6%)	4 (12.5%)	0.719^b^

^a^Data are expressed as the means ± standard deviations using paired sample rank sum test

^b^*X*^2^ test *P* < 0.05 was considered statistically significance

## Discussion

Pregnancy is a special period for women, and the need for thyroid hormones differs significantly from non-pregnant status. When the body’s demand for thyroid hormone is not met, it may lead to the occurrence of hypothyroidism during pregnancy. If SCH cannot be corrected during pregnancy, which can lead to a variety of adverse pregnancy outcomes, such as spontaneous abortion, preeclampsia, gestational hypertension, gestational diabetes, preterm birth, and reduced IQ of offspring [18, 19]. SIBO affects the absorption of thyroid drugs and aggravates hypothyroidism during pregnancy [20]. Therefore, early clinical intervention is particularly important for patients with hypothyroidism during pregnancy, especially those with SIBO.

In this study, we found that the positive rate of methane-hydrogen in pregnant women with hypothyroidism during pregnancy was higher than that in normal pregnant women, which is consistent with the results of Wang et al. [21]. We analyze possible reasons: (1) thyroid hormone regulates bowel motility [20], and hypothyroidism during pregnancy is associated with a decrease in the frequency of intestinal activity. Reduced intestinal motility provides the conditions for bacterial overgrowth in small intestinal segments. (2) Thyroid hormone slows intestinal motility by reducing 5-hydroxytryptamine (5-HT) to inhibit the contraction of the migration motor complex, which reduces its ability to clear intestinal bacteria and promotes SIBO [21, 22].

In this study, the total score of GSRS, average score of dyspepsia syndrome and average score of constipation syndrome of SCH pregnant women were higher than those of normal pregnant women, and the positive rate of methane was also higher than that of normal pregnant women. As early as 2010, Ebert [23] indicated that constipation was more common in patients with hypothyroidism. In 2018, Takakura and Pimentel’s study [24] showed that methane can directly reduce intestinal transport leading to constipation. We analyze the possible reasons as follows: (1) disruption of the intestinal barrier can cause loss of nutrients in the gut, the energy needed to reduce the intestinal smooth muscle cell contraction, reduced intestinal motility; (2) pressure on the bowel by an enlarged uterus during pregnancy may result in reduced bowel motility; (3) regulation of peristalsis of thyroid hormone is reduced in the SCH condition [20]; (4) methanogenesis in methane^+^ amplifies neuronal activity through the cholinergic pathway, leading to small intestinal dyskinesia [25].

This study found that the hsCRP level of the subclinical hypothyroidism group was higher than that of the normal group of pregnant women, which was consistent with the high hsCRP level in patients with SCH indicated by the meta-analysis of Tellechea [26] TSH can increase the secretion of interleukin-6 (IL-6), the main inducer of CRP, by upregulating the promoter activity of the IL-6 gene and increasing the stability of its mRNA of Raychaudhuri et al. [27]. Female patients with subclinical hypothyroidism have higher levels of IL-6 [28], which has been confirmed in previous studies.IL-6 can damage the tight junctions of epithelial cells and disrupt the intestinal barrier.

The average methane and hydrogen abundances in the SCH group in this study were higher than those in the control group. A previous study by our group found that the intestinal flora of pregnant women with hypothyroidism had higher levels of Gammaproteobacteria and Prevotella than normal pregnant women [29]. Previous studies have shown that Gammaproteobacteria and Prevotella are positively correlated with hydrogen production [30, 31], and we speculate that this is why the average hydrogen abundance in the hypothyroidism group is higher than that in the normal group. The 2020 ACG guidelines pointed out that *Methanobrevibacter smithii* [32], an archaea that produces methane in the small intestine, uses hydrogen as a substrate to produce methane. We speculated that the high methane abundance might be due to the elevated hydrogen-producing bacteria in the small intestine, providing a substrate for *Methanobrevibacter smithii*. However, there are few studies on *Methanobrevibacter smithii* archaea in SIBO patients, and this will be a focus of our next study.

In this study, The TSH of thyroid function in the intervention group was lower than that before treatment, and the level of thyroid function was stable below the target value of treatment. Talebi et al. [9] found that supplementation of probiotics and prebiotics on the basis of levothyroxine could significantly reduce the level of TSH, which was consistent with our results. This shows that, for patients with well-controlled thyroid function in the second trimester, the combined application of probiotics and prebiotics is beneficial to maintain the stability of thyroid function. Levothyroxine sodium is absorbed mainly in the small intestine [33], and as mentioned above, SIBO status disrupts the intestinal barrier, and we hypothesized that probiotics and prebiotics may improve the intestinal barrier and promote the absorption of LT_4_. Second, gut bacteria can express β-glucuronidase and sulfatase to inactivate thyroid hormones in the liver [9], and study has shown that *Lactobacillus acidophilus* can significantly reduce the β-glucuronidase activity in feces [34]. Therefore, we hypothesized that *Lactobacillus acidophilus* in quadruple probiotics may reduce thyroid hormone inactivation by down-regulating intestinal microorganisms expressing β-glucuronidase.

This study showed that TC, TG, and LDL were decreased, and HDL levels were increased after treatment with probiotics and prebiotics. Rajkumar et al. [35] showed in their study on the treatment of obese people with probiotics that probiotics can reduce TC, TG, and LDL and increase HDL, which is consistent with our study results. Studies have shown that the lipid metabolite phosphatidylethanolamine (PE) in SCH pregnant women can promote inflammatory reaction through the pathogenic bacillus infection response pathway, leading to dyslipidemia. The addition of probiotics may correct the dyslipidemia by down-regulating PE [36–38]. Probiotics may also lower cholesterol by reducing the enterohepatic circulation of bile acids and activating thyroid hormones in brown fat and muscle [39]. We speculate that the combination of probiotics and prebiotics can reduce dyslipidemia by downregulating PE, reducing enterohepatic circulation of bile acids, and mobilizing thyroid hormones.

After 21 days of treatment with probiotics (quadruple probiotics) + prebiotics (polysaccharide fiber powder), 28.1% of in intervention group turned negative and positive rate of methane, hsCRP level, GSRS scale total score, diarrhea syndrome, indigestion syndrome, and constipation syndrome score were lower than those before treatment and also the abundance of hydrogen and methane in the intervention group after treatment lower than before. Zheng et al.’s study [40] that probiotics and prebiotics can significantly reduce CRP level and Zhong et al.’s study [41] that probiotics have a significant effect on relieving SIBO symptoms are consistent with the results of this study. We consider that probiotics plus prebiotics may treat SIBO in the following ways: (1) regulation of intestinal motility: probiotic strains can mediate host-tissue interactions, increase the tight junctions of intestinal epithelial cells, improve the integrity of the small intestinal barrier [42], regulate intestinal movement, and correct clinical symptoms such as diarrhea and constipation. (2) improve the intestinal barrier: The study by Frei et al. [43] showed that the combined application of probiotics and prebiotics can increase the regulatory T cells (Treg cells) that suppress the inflammatory response and increase the antibacterial protein through the PRR-MAMP pathway. Together, they promote the formation of the mucosal barrier. In addition, studies have shown that lactic acid produced by *Lactobacillus acidophilus* among probiotics [44, 45], short-chain fatty acids (SCFAs) produced by *Enterococcus faecalis* and prebiotics, organic acids produced by Bifidobacterium and Lactobacillus [13] and antibacterial compounds that may improve the intestinal barrier and increase the formation of lysozyme, inhibit the growth of pathogens and stabilize the small intestinal barrier. In addition, we will continue to treat pregnant women who have not turned negative in this course of lactulose methan-hydrogen breath test until they turn negative.

At present, there are few studies on SCH in pregnancy combined with small intestinal bacterial overgrowth about treatment. This study can provide new ideas for the treatment of subclinical hypothyroidism in pregnancy combined with SIBO. However, this study still has some limitations. With our small sample size, pregnancy outcomes in pregnant women with hypothyroidism and small intestinal bacterial overgrowth have not been longitudinally tracked.

In conclusion, we found a strong association between small intestinal bacterial overgrowth and subclinical hypothyroidism in pregnancy. Probiotics (quadruple probiotics) combined with prebiotics (polysaccharide fiber powder) have a good effect on improving small intestinal bacterial overgrowth in patients with subclinical hypothyroidism during pregnancy. Their combined use can promote the stabilization of thyroid function and correct small intestinal bacterial overgrowth. This provides a new path for the treatment of pregnant women with small intestinal bacterial overgrowth.

## Data Availability

The raw data supporting the conclusions of this article will be made available by the authors, without undue reservation.
